# Immediate versus delayed quitting and rates of relapse among smokers treated successfully with varenicline, bupropion SR or placebo

**DOI:** 10.1111/j.1360-0443.2010.03058.x

**Published:** 2010-11

**Authors:** David Gonzales, Douglas E Jorenby, Thomas H Brandon, Carmen Arteaga, Theodore C Lee

**Affiliations:** 1 OHSU Smoking Cessation Center, Department of Medicine, Oregon Health & Science UniversityPortland, OR, USA; 2 University of Wisconsin School of Medicine and Public HealthMadison, WI, USA; 3 H. Lee Moffitt Cancer Center and Research InstituteTampa, FL, USA; 4 Pfizer Inc.New York, NY, USA

**Keywords:** Bupropion sustained-release, lapse recovery, quitting patterns, smoking cessation, varenicline

## Abstract

**Aims:**

We assessed to what degree smokers who fail to quit on the target quit date (TQD) or lapse following TQD eventually achieve success with continued treatment.

**Design:**

A secondary analysis of pooled data of successful quitters treated with varenicline (306 of 696), bupropion (199 of 671) and placebo (121 of 685) from two identically-designed clinical trials of varenicline versus bupropion sustained-release and placebo.

**Setting:**

Multiple research centers in the US.

**Participants:**

Adult smokers (*n* = 2052) randomized to 12 weeks drug treatment plus 40 weeks follow-up.

**Measurement:**

The primary end-point for the trials was continuous abstinence for weeks 9–12. TQD was day 8. Two patterns of successful quitting were identified. Immediate quitters (IQs) were continuously abstinent for weeks 2–12. Delayed quitters (DQs) smoked during 1 or more weeks for weeks 2–8.

**Findings:**

Cumulative continuous abstinence (IQs + DQs) increased for all treatments during weeks 3–8. Overall IQs and DQs for varenicline were (24%; 20%) versus bupropion (18.0%, *P =*0.007; 11.6%, *P <*0.001) or placebo (10.2%, *P <*0.001; 7.5%, *P <*0.001). However, DQs as a proportion of successful quitters was similar for all treatments (varenicline 45%; bupropion 39%; placebo 42%) and accounted for approximately one-third of those remaining continuously abstinent for weeks 9–52. No gender differences were observed by quit pattern. Post-treatment relapse was similar across groups.

**Conclusions:**

Our data support continuing cessation treatments without interruption for smokers motivated to remain in the quitting process despite lack of success early in the treatment.

## INTRODUCTION

Cigarette smoking remains a critical public health problem, resulting in unnecessary illness and deaths world-wide [1]. While effective smoking cessation treatments, including counseling, social support and pharmacotherapies [1], are widely available, quitting success rates can vary significantly [2]. Even among those who receive evidence-based treatments, adherence to recommendations is often poor, with negative implications for treatment outcomes [3,4], suggesting the importance of longer-term adherence support for those patients attempting actively to quit smoking.

Adverse events, fear of becoming dependent upon medication and cost have all been associated with premature discontinuation of pharmacological treatments [5,6]. An area receiving more recent attention has to do with the role played by different definitions of quitting success or abstinence in treatment outcomes [2,7,8]. Definitions of treatment failure linked to the target quit day (TQD) [9,10] may contribute to interruption of an active quitting process [3,7], when smokers believe they have already failed due to inability to achieve initial abstinence or maintain continuous abstinence after the planned TQD. Additionally, drug labeling that cautions patients about risks of smoking while using nicotine replacement therapy (NRT) [3,11,12], and some health insurance guidelines such as those of the National Institute for Health and Clinical Excellence (NICE) in the United Kingdom, that assess quit success at 4 weeks after the TQD [13], may have the unintended effect of prematurely shortening the duration of treatment for those not abstinent at 4 weeks. This is despite evidence supporting enhanced efficacy with better adherence to recommended duration of therapy [1,6,14].

No uniform standard has been established for when ‘smoking abstinence’ should be assessed [2]. The definitions of abstinence can vary considerably [2], including 24 hours [8,10,15,16], 4 weeks following a TQD [17] and the last 4 weeks of treatment [18], as well as other intervals [2,7].

The definition of abstinence employed can result in decisions to change, interrupt or discontinue treatments, as has been suggested for those who fail to quit on, or shortly after, a TQD [9,10]. In an effort to guide more uniformity and flexibility when treating smokers, the Society of Research on Nicotine and Tobacco (SRNT) recommended including a 2-week ‘grace period following the TQD when assessing successful abstinence’[18]. However, successful quitting in clinical practice and much of the literature remains focused upon abstinence end-points [7,8], while much remains unknown about quitting and smoking behaviors [19]. Quitting patterns and processes during a quit attempt is an under-investigated but emerging area of research [7,8,20]. Previous research has not established the degree to which motivated smokers who fail to achieve immediate abstinence on the recommended TQD, or who experience early lapses, would eventually achieve continuous abstinence if treatment were not interrupted or discontinued. Secondary analyses of data from drug efficacy trials can expand our knowledge of quitting processes beyond the TQD. Although these trials are focused upon abstinence end-points, often the last 4 weeks of treatment [8,18], abstinence data are collected beginning with the TQD. All participants, regardless of abstinence status, are encouraged to take the study drug, to continue in their attempts to achieve or maintain abstinence and to remain actively engaged for the entire treatment period [21,22]. The result is a rich database for analyses of quitting patterns throughout the treatment period and across various treatment types.

To investigate successful quitting patterns among all those achieving continuous abstinence for weeks 9–12, a *post hoc* analysis was conducted on pooled data from two identical varenicline versus bupropion sustained-release (SR) and placebo randomized controlled trials (RCTs). Our purpose was to examine the contribution of two subgroups of successful quitters who achieved continuous abstinence for at least the last 4 weeks of treatment. One group quit on the TQD and remained continuously abstinent throughout the 12-week treatment period. The other group had periods of smoking prior to attaining continuous abstinence for at least the last 4 weeks. In addition to examining overall patterns of successful quitting, we tested two primary hypotheses with respect to differential medication effects. First, because varenicline's partial agonist and antagonist activity at α4β2 receptors has been reported to be linked to reduction in pleasure and reward from smoking [21,22], we hypothesized that quitting patterns for participants in the varenicline arm may be more dynamic across the 12 weeks of treatment compared with participants in the bupropion SR or placebo (counseling alone) arms. That is, we expected that smokers unable to achieve abstinence on the TQD or who experienced early lapses would be more likely to recover if they were in the varenicline arm. Therefore, we predicted that the varenicline arm would have a higher proportion of delayed quitters than the other two arms. Secondly, we hypothesized that the experience of reduced rewards when smoking while taking varenicline may blunt motivation to return to smoking and provide some protection from relapse post-drug treatment.

## METHODS

### Setting and participants

The overall design and methodology of these trials have been described previously in full in published primary manuscripts [21,22].

Briefly, both studies were identically designed randomized, double-blind, placebo-controlled trials conducted between June 2003 and April 2005. Participants were generally healthy adult smokers. Those with any history of bupropion or varenicline exposure were excluded to reduce risk of re-treatment bias [23].

### Interventions

Participants in each study were randomized at baseline to receive varenicline, bupropion or placebo for 12 weeks. All participants were provided with a self-help booklet on smoking cessation (*Clearing the Air: How to Quit Smoking*[24]) at baseline. The TQD followed the first week of drug treatment and was day 8 (week 1 visit). These were placebo-controlled trials with respect to drug assignments, but all arms, including placebo, included brief cessation counseling (up to 10 minutes) [25] at baseline and clinic visits for weeks 1 to 13, 24, 36, 44 and 52. Brief (5-minute) telephone counseling calls were scheduled for day 3 after the TQD and at weeks 16, 20, 28, 32, 40 and 48 during the non-treatment follow-up. Smoking status was assessed at clinic visits during active treatment (weeks 1–12) and the non-drug treatment follow-up phase (weeks 13, 24, 36, 44 and 52). Expired carbon monoxide (CO) was measured during clinic visits to confirm smoking status.

### Outcomes

The primary end-point of both trials was continuous abstinence (not even a puff) for weeks 9–12, and confirmed by CO levels ≤10 parts per million (p.p.m.) at clinic visits. A secondary end-point was continuous abstinence for weeks 9–52 confirmed at in-clinic visits.

Pooled analyses of overall efficacy data have been reported previously [26]. This *post hoc* analysis of quitting patterns of pooled data for successful quitters is the first to be conducted. Successful quitters were defined as those who met the criteria of continuous abstinence for the primary end-points (weeks 9–12). Successful quitters were classified further as either ‘immediate quitters’ (IQs) or ‘delayed quitters’ (DQs). IQs achieved initial abstinence immediately on the day 8 TQD and remained continuously abstinent for weeks 2–12. The term DQs was used to categorize all those who first quit later than their TQD, as well as those who quit on schedule, but smoked in a subsequent week(s), and were then able to achieve continuous abstinence for weeks 9–12. Thus, DQs includes all those who were ‘delayed’ in successfully achieving continuous abstinence for the primary end-point: weeks 9–12.

### Analysis

All analyses were conducted on pooled data from the two studies. Analyses of continuous abstinence for weeks 9–12 and weeks 9–52 were conducted for the two quitting patterns. Continuous abstinence rates (IQs + DQs) were analyzed weekly for weeks 2–12 to assess cumulative rates of continuous abstinence. The rates of relapse for IQs and DQs were assessed during the non-treatment follow-up at weeks 13, 24, 36, 44 and 52 and compared across all three treatment arms (varenicline, bupropion SR and placebo) based on the sample of all successful end-of-treatment quitters and by quitting pattern (IQs and DQs). In addition, for the continuous abstinence for weeks 9–52, the interaction between treatment arms and quitting pattern was investigated.

For the primary and secondary end-points, analyses to assess treatment effects were performed using logistic regression models with treatment group and study as the main effects. Hypotheses were tested using two-tailed likelihood-ratio χ^2^ tests with a significance level of 0.05. Odds ratios (OR) and 95% confidence intervals (CI) for continuous abstinence rates were calculated.

For the continuous abstinence for weeks 9–52, the interaction effect between treatment arms and quitting pattern was assessed using a logistic regression model, including the main effects of treatment, study and quitting pattern and the treatment × quitting pattern interaction.

To assess comparability across treatment groups, demographics and baseline characteristics were summarized by treatment group for the pooled all-randomized sample and for each quitting pattern subsample.

All analyses were performed using SAS version 8 in a UNIX platform.

## RESULTS

### Participant disposition

**Figure 1:**
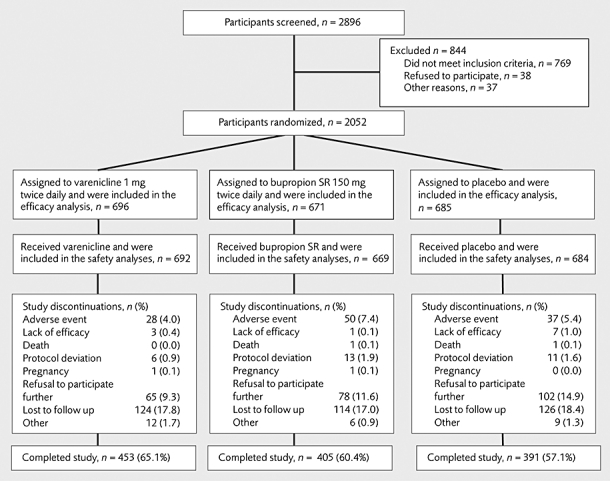
Participant disposition

Of the 2052 randomized participants from the two trials (Fig. 1), those meeting the criteria for successful quitters were 306 of 696 for varenicline, 199 of 671 for bupropion and 121 of 685 for placebo.

Baseline characteristics of the all-randomized sample and the subsamples of successful quitters (IQs and DQs) for each of the treatment groups are shown in Table 1. The mean baseline Fagerström Test for Nicotine Dependence (FTND) [27] scores were lower in the IQs and DQs than in their corresponding all-randomized treatment group sample, except for the bupropion DQ group. No gender differences by quitting pattern were observed. Demographic characteristics and smoking histories of the overall sample were generally comparable across treatment groups (Table 1).

**Table 1:** Baseline characteristics.

				***Successful quitters***					
	*All randomized*			*Immediate quitters*			*Delayed quitters*		
	*Varenicline (n = 696)*	*Bupropion SR (n = 671)*	*Placebo (n = 685)*	*Varenicline (n = 167)*	*Bupropion SR (n = 121)*	*Placebo (n = 70)*	*Varenicline (n = 139)*	*Bupropion SR (n = 78)*	*Placebo (n = 51)*
Mean age, years (range)	43.5 (18–75)	42.5 (18–75)	42.5 (18–75)	45.7 (19–72)	45.4 (21–74)	44.2 (23–74)	44.5 (19–71)	44.9 (19–73)	44.4 (19–72)
Gender, *n* (%)									
Men	366 (52.6)	398 (59.3)	384 (56.1)	85 (50.9)	73 (60.3)	44 (62.9)	73 (52.5)	48 (61.5)	25 (49.0)
Women	330 (47.4)	273 (40.7)	301 (43.9)	82 (49.1)	48 (39.7)	26 (37.1)	66 (47.5)	30 (38.5)	26 (51.0)
Race, *n* (%)									
White	574 (82.5)	547 (81.5)	552 (80.6)	153 (91.6)	108 (89.3)	62 (88.6)	121 (87.1)	58 (74.4)	39 (76.5)
Black	67 (9.6)	64 (9.5)	75 (10.9)	4 (2.4)	5 (4.1)	3 (4.3)	8 (5.8)	10 (12.8)	9 (17.6)
Asian	12 (1.7)	9 (1.3)	15 (2.2)	1 (0.6)	2 (1.7)	1 (1.4)	1 (0.7)	–	1 (2.0)
Other	43 (6.2)	51 (7.6)	43 (6.3)	9 (5.4)	6 (5.0)	4 (5.7)	9 (6.5)	10 (12.8)	2 (3.9)
No. of years smoked									
*n*	695	671	684	167	121	70	139	78	51
Mean (range)	25.7 (2–59)	24.8 (2–61)	24.6 (0–61)	27.3 (3–58)	25.8 (4–57)	25.9 (5–55)	27.2 (4–51)	26.3 (3–55)	25.0 (2–56)
No. of cigarettes per day in past month									
*n*	695	671	684	167	121	70	139	78	51
Mean (range)	21.8 (10–70)	21.4 (10–65)	21.5 (10–80)	20.4 (10–60)	19.5 (10–40)	20.7 (10–40)	21.2 (10–67)	21.6 (10–50)	18.7 (10–40)
Baseline FTND score^a^									
*n*	693	670	681	166	121	69	138	78	51
Mean (range)	5.28 (0–10)	5.29 (0–10)	5.27 (0–10)	4.81 (0–10)	4.51 (0–9)	4.86 (0–10)	4.92 (0–9)	5.26 (0–9)	4.47 (0–10)
≥1 prior quit attempt *n/N* (%)	585/695 (84.2)	576/671 (85.8)	578/684 (84.5)	148/167 (88.6)	110/121 (90.9)	63/70 (90.0)	121/139 (87.1)	67/78 (85.9)	43/51 (84.3)

FTND: Fagerström Test for Nicotine Dependence.

a Range 0–10; higher scores indicate greater dependence [27].

### Patterns of successful quitting during the drug treatment period: immediate and delayed

**Figure 2:**
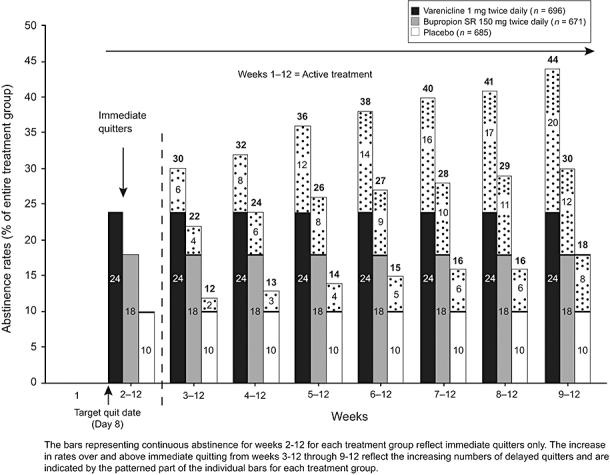
Cumulative contributions of immediate quitters (IQs) and delayed quitters (DQs) to continuous abstinence rates, week X to week 12. Varenicline versus bupropion SR IQs (24.0%, 18.0%; *P*= 0.007); DQs (20.0%, 11.6%; *P*< 0.001). Varenicline versus placebo IQs (24.0%, 10.2%; *P*< 0.001); DQs (20.0%, 7.5%; *P* < 0.001). Bupropion SR versus placebo IQs (18.0%, 10.2%; *P*< 0.001) and DQs (11.6%, 7.5%; *P*= 0.009)

Successful quitting (continuous abstinence for weeks 9–12) included IQs (continuously abstinent weeks 2–12) and DQs (smoked at 1 or more weeks for weeks 2–8). The cumulative rates of continuous abstinence increased with each week of treatment up to weeks 9–12, regardless of type of treatment (Fig. 2).

Overall, a significantly greater percentage of the total randomized varenicline participants compared with bupropion SR participants were IQs (24.0% versus 18.0%, *P* = 0.007) and DQs (20.0% versus 11.6%, *P* < 0.001). This was also true of varenicline versus placebo (IQs, 24.0% versus 10.2%, *P* < 0.001; DQs, 20.0% versus 7.5%, *P* < 0.001). There were also significantly greater percentages of IQs and DQs in the bupropion SR group than the placebo group (IQs, 18.0% versus 10.2%, *P* < 0.001; DQs, 11.6% versus 7.5%, *P* = 0.009).

**Figure 3:**
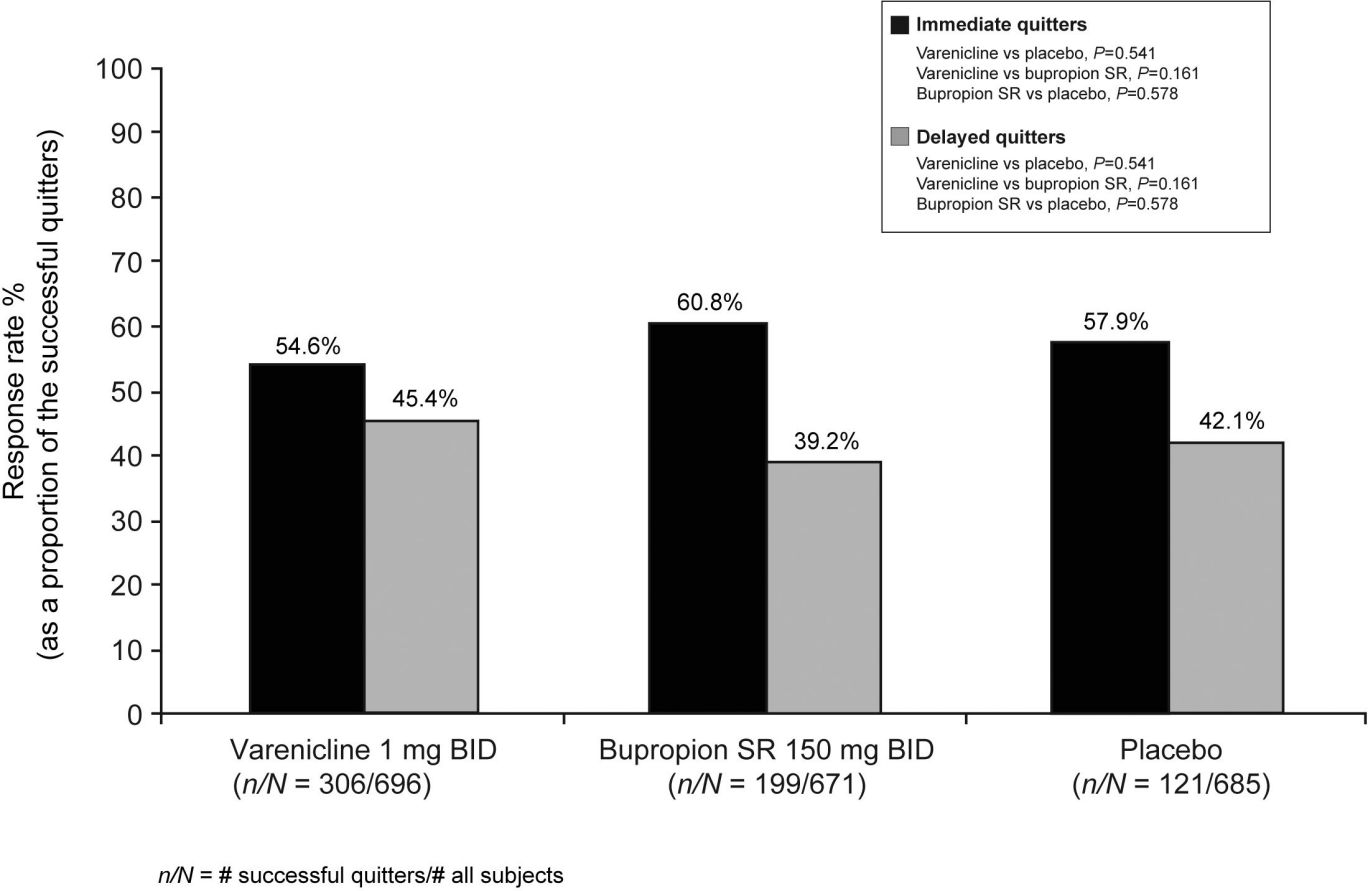
Continuous abstinence rates, weeks 9–12, by quit pattern, immediate or delayed. Differences between treatment groups were not significant

Analysis with ‘successful quitters only’ as the denominator revealed that DQs as a proportion of successful quitters was similar across the three treatment arms. Forty-five per cent of successful varenicline quitters were DQs versus 39% for bupropion SR (*P*= 0.161) and 42% for placebo (*P*= 0.541). None of the differences in proportions were statistically significant (Fig. 3).

**Figure 4:**
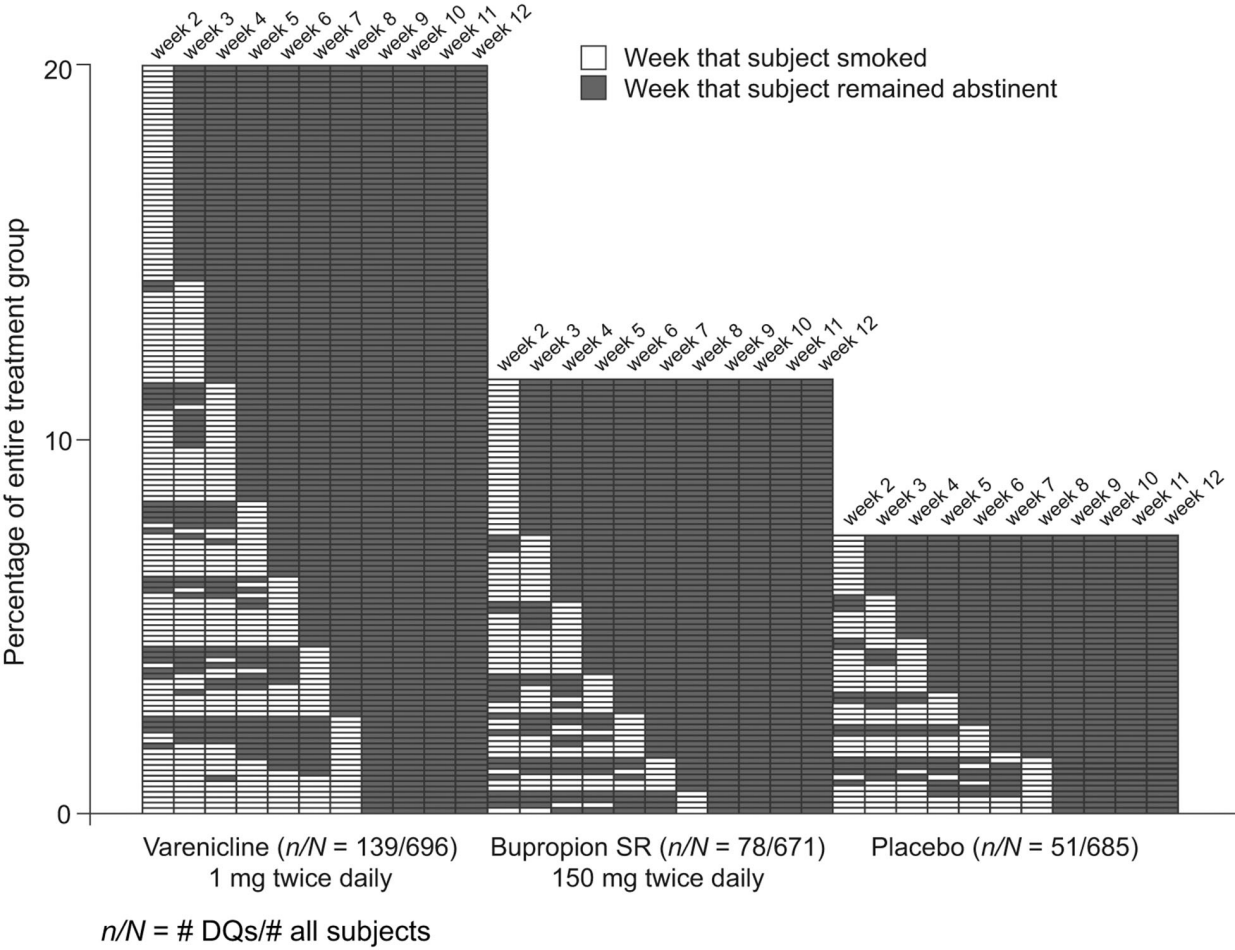
Weekly abstinence status of delayed quitters (DQs) following the day 8 TQD weeks 2–12. Most DQs remained continuously abstinent from the point of the first week of abstinence and did not lapse. DQs who lapsed generally re-established abstinence in the week following the lapse

Abstinence status for each individual DQ participant for the weeks following the TQD (weeks 2–12) are presented in Fig. 4.

### Post-treatment abstinence

Results from the logistic regression analysis (based on the total number of participants who were continuously abstinent from weeks 9 to 12) showed a statistically significant (*P* = 0.001) effect of quitting pattern on continuous abstinence to week 52, with DQs less likely to remain abstinent. However, treatment effect was not statistically significant (*P* = 0.782 varenicline versus placebo; *P* = 0.983 varenicline versus buproprion SR; *P* = 0.784 buproprion versus placebo), nor was the interaction between treatment arm and quitting pattern (*P* = 0.239). DQs made up approximately one-third of the participants who remained continuously abstinent from weeks 9 to 52 regardless of treatment group.

### Rates of relapse (decline in continuous abstinence)

**Figure 5:**
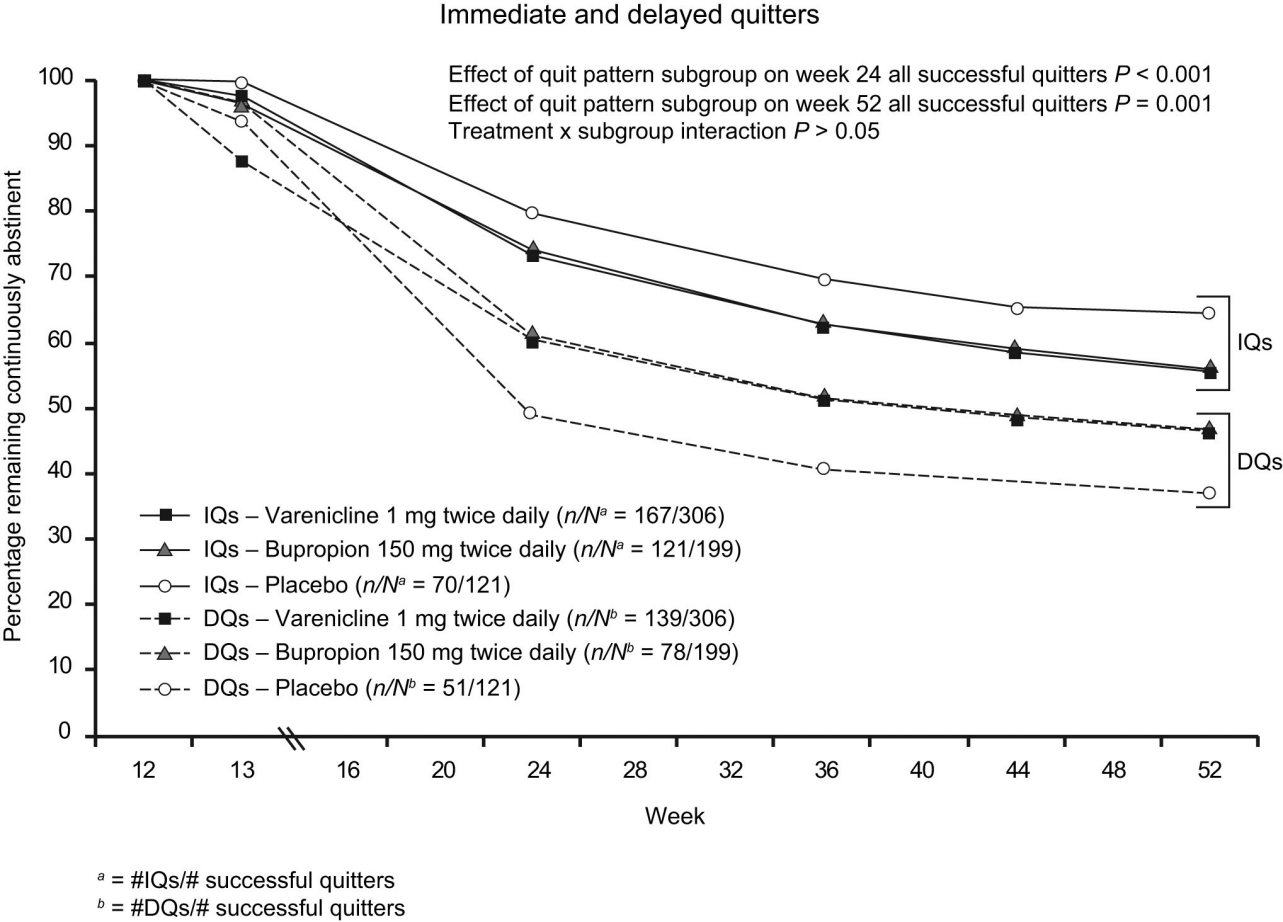
Post-treatment relapse (rate of decline in continuous abstinence) by quit pattern (weeks)

Analysis using the total number of participants who were continuously abstinent from weeks 9 to 12 in each treatment group in the denominator shows that the relative rate of decline in continuous abstinence following the end-of-treatment to week 52 is similar for each treatment group (Fig. 5). There was no significant difference between the relapse rates at week 24 and week 52 across treatment groups. There was a significant effect of quit pattern subgroups (IQs versus DQs) on week 24 and week 52 continuous abstinence (*P*≤ 0.001, Fig. 5). There was no treatment × subgroup interaction at either week 24 (*P* = 0.159) or week 52 (*P* = 0.239).

## DISCUSSION

In this secondary analysis of pooled data from two identical varenicline versus bupropion and placebo trials, two successful quitting patterns were identified among smokers who achieved continuous abstinence for the last 4 weeks of treatment (weeks 9–12). Immediate quitters (IQs) quit on the TQD (day 8) and remained continuously abstinent for weeks 2–12. Delayed quitters (DQs) achieved initial abstinence some time after the TQD or may have lapsed following abstinence at week 2 and recovered by week 9 of the trial. Compared to IQs the DQs were ‘delayed’ in achieving continuous abstinence to the end-of-treatment.

Although varenicline produced a greater total number of abstinent participants for weeks 9–12, our first hypothesis that quitting might be more dynamic for smokers treated with varenicline was not supported. Two unexpected findings from our analyses were that IQ and DQ patterns were similar regardless of treatment group, and that the majority of DQs remained continuously abstinent to the end-of-treatment, following their first reported week of no smoking. Most did not lapse (Fig. 4).

Our data expand upon findings from recent studies of quitting processes and patterns and suggest that IQ and DQ may be natural patterns in treatment-seeking smokers, regardless of therapy. In one report self-quitters who intended to quit abruptly (IQ) were often unable to do so on the TQD, but continued to work towards initial abstinence [7]. NRT-treated smokers who continued treatment despite lapsing also showed a pattern of increasing abstinence over time [8]. Both studies described quitting smoking as a dynamic process that could extend beyond the TQD and suggested that intervening following a lapse could reduce risk of complete relapse. No special intervention was provided for our DQs to help with either achieving initial continuous abstinence or with lapse recovery. For many smokers who could become successful DQs, adhering to the planned treatment duration may be a sufficient intervention to improve abstinence outcomes. Collectively, ours and the other emerging data have begun to demonstrate that quitting smoking is a more complex and dynamic process than previously understood, and one that can extend beyond the TQD and persist for several weeks [7,8,20].

Previously published reports from these trials indicated that varenicline-treated participants had significantly greater reductions in some of the reinforcing effects of smoking such as smoking satisfaction, psychological reward and pleasure [21,22]. These observations and others that reported enjoyment of smoking as a barrier to quit attempts [19] led to our second hypothesis that varenicline may provide some protection from post-treatment relapse in successful quitters. Because participants were not encouraged to abstain from smoking prior to the TQD (day 8 of treatment), all had some experience with smoking combined with the effects of varenicline. We thought that motivation to return to smoking after the end-of-treatment might be blunted due to recently experienced blunted effects when smoking while taking varenicline. This hypothesis was not supported. Varenicline did not provide additional protection against relapse post-treatment. Instead, we found that post-treatment relapse rates were nearly identical for successful quitters in all treatment groups and by quitting pattern. Even though DQs in all groups made up a substantial portion of those who remained continuously abstinent to week 52, they experienced more post-treatment relapse than IQs. These higher relapse rates suggest that a smoker's ability to abstain from smoking on the TQD and to remain abstinent during treatment is also linked to the maintenance of long-term abstinence. At first glance, this finding appears consistent with earlier research showing that any smoking after the TQD predicts poorer outcomes [9]. However, as we and others have shown, successful quitting processes are more complex than can be assessed adequately shortly after the TQD or based on lapses [7,8,20].

Recent analyses from a varenicline relapse prevention trial suggested that extending treatment beyond the standard 12 weeks would be especially helpful for quitters who achieved initial abstinence after the TQD [20]. Previous data published from that trial [28] and a bupropion relapse prevention trial [29] provided the basis for allowing up to 24 weeks of treatment being included in the package inserts [17,30]. Nicotine dependence is a chronic condition and post-treatment relapse is common [1]. Identification of which successful quitters might be more likely to benefit from an extended period of treatment to prevent relapse could help guide the clinician's treatment decisions. A recent analysis of data from these trials suggests that those continuously abstinent from TQD were less likely to relapse than those abstinent only for weeks 9–12 [31]. We speculate that DQs may be more likely to benefit from additional weeks of treatment, but this has not been tested directly. Counseling post-12 weeks may also aid in preventing relapse.

This is the first analysis that we are aware of that investigates the quitting patterns of smokers treated with varenicline, bupropion or counseling alone (placebo) who fail to quit on a TQD, or lapse, who go on to become successful end-of-treatment quitters. There are several important features of the studies from which the data were analyzed. These were two large randomized placebo-controlled trials that included a head-to-head comparison of two smoking cessation drugs and placebo. Because the same level of brief cessation counseling was provided in all arms, the placebo arm could be considered a surrogate for ‘counseling alone’. Overall, treatment completion rates were consistent with prior trials of bupropion [32,33].

There are limitations to our analyses that may limit interpretation of the results for a broader population of smokers. This was a *post hoc* analysis of quitting patterns for successful end-of-treatment quitters. Unsuccessful quitting patterns were not assessed. We felt that analyzing patterns of successful quitters could have more immediate practical relevance to clinical practice and there have been other ‘quitters only’ analyses reported in the literature [34]. Investigation of patterns for unsuccessful quitters would be an important next step to expanding our understanding of quitting patterns overall.

In addition, participants were generally healthy, motivated to quit smoking and received up to 10 minutes of face-to-face cessation counseling every week during treatment and at clinic visits during post-drug follow-up. Counseling treatments available outside clinical trials that provide fewer or briefer sessions may result in poorer abstinence outcomes, and proportions of DQs may vary. Lastly, treatment with NRT was not part of the study design, and quitting patterns for 12 weeks of NRT treatment may vary from the two patterns identified in our analyses.

Our data and those from other recent studies offer a more optimistic picture about the potential effects of pharmacological and behavioral treatment following lapses [8] and failures to quit on the TQD [7]. By adjusting the definition of treatment success so that failure-to-quit on the TQD [9,10], or lapsing [35], are not regarded as treatment failure and persisting with treatment over the entire recommended period, it seems likely that more smokers could be successful even without additional relapse prevention interventions. However, there are some challenges to adopting this newer approach. Revisions to product labeling to encourage continuation of treatment more directly may be needed. Currently, labels for NRT in many countries warn patients not to smoke while using NRT and to consider postponing their efforts to quit by the fourth week of treatment, if abstinence has not been achieved [11,12]. The label for bupropion SR may discourage continued treatment and delayed quitting by indicating that smoking after a quit date significantly reduces chance of success [30]. The label for varenicline that encourages patients to continue treatment and continue attempting to achieve abstinence despite lapses [17] may be a good model to support delayed quitting. Clinicians and tobacco treatment specialists would need to adjust messaging to patients and revise treatment protocols to support continuation of treatment. Lastly, health insurance benefit coverage for smoking cessation treatment and government guidelines regarding treatment success can play a role. For example, the NICE guidelines assess cessation success at 4 weeks following the TQD [13]. Our data suggest that assessing success at a later point might result in capturing additional quitters.

In summary, an important question for clinicians to consider in smoking cessation treatment is whether or not to modify, interrupt or continue a specific treatment for motivated smokers who fail to achieve or to maintain continuous abstinence following a planned TQD. Previous studies have not reported sufficient evidence to guide these decisions. Our data show that among quitters who completed any of the treatments, a substantial proportion failed to achieve abstinence on the planned TQD or had lapses prior to quitting successfully. Had treatment been interrupted or discontinued for these ‘delayed quitters’, opportunities for achieving continuous abstinence could have been lost for up to 45% of successful quitters. These data support recommending continuing cessation treatments without interruption for smokers motivated to remain in the quitting process despite lack of success early in treatment.

## Clinical trial registration

Clinicaltrials.gov Identifier: NCT00141206 and NCT00143364
